# Special Issue ‘Food Fermentations: Microorganisms in Food Production and Preservation’: Editorial

**DOI:** 10.3390/microorganisms11030569

**Published:** 2023-02-24

**Authors:** Spiros Paramithiotis

**Affiliations:** Laboratory of Food Process Engineering, Department of Food Science and Human Nutrition, Agricultural University of Athens, 75 Iera Odos St., 11855 Athens, Greece; sdp@aua.gr

For centuries, microorganisms have been exploited for the production and preservation of substates intended for human consumption. The geospatial and seasonal availability of raw materials of plant and animal origin, microclimatic conditions, and the processing steps that have been employed to facilitate specific bioconversions have resulted in the development of a wide range of fermented products. The qualitative and quantitative composition of the microecosystem of raw materials, along with the effect of intrinsic, environmental, biotic and abiotic factors on the microbial dynamics, chemical composition and properties of the final products, have been extensively assessed over the last few decades.

Effective assessment of spontaneously driven food fermentations essentially involves three steps. The first step is to analyze the microcommunity that drives the fermentation, the dynamics of the microorganisms that constitute it and the factors that direct it. The methodology that is currently available includes both culture-dependent and -independent approaches, the combination of which may successfully delineate the composition of the microecosystem, down to sub-species level. The second step is the evaluation of the potential of the microorganisms participating in these microcommunities. For that purpose, a series of properties related to the development, safety and functionality of the final product is considered. This assessment can be effectively carried out through whole-genome sequencing and comparative genomic analysis of the isolates under consideration, complemented with phenotypic verification. Finally, the third step is to verify the estimated potential of the most interesting microbial strains through their utilization under laboratory and ultimately under actual production conditions.

This Special Issue is honored to include five excellent contributions, which cover all the aforementioned steps. Huang et al. [1] combined shotgun metagenomic sequencing and metabolomics in order to assess the composition and functions of the microcommunity developed during light-flavor Baijiu fermentation. Through the combination of these approaches, it was possible to assign starch saccharification primarily to *Lichtheimia ramosa*, *Saccharomycopsis fibuligera* and *Bacillus licheniformis*, ethanol production to *Saccharomyces cerevisiae* and *Pichia kudriavzevii* and production of flavor-associated metabolites to members of the former-*Lactobacillus* genus, such as *Lactiplantibacillus plantarum*, *Levilactobacillus brevis* and *Secundilactobacillus odoratitofui*.

Two studies evaluated the capacity of microorganisms to serve as starter cultures towards the production of commodities with specific characteristics. Syrokou et al. [2] performed a series of phenotypic tests to evaluate the technological and safety attributes of 195 yeast and 207 lactic acid bacteria isolates retrieved from spontaneously fermented Greek wheat sourdoughs. The proteolytic, lipolytic and antimicrobial capacities of several yeast and LAB strains were reported and further studied. Finally, two *Lp. plantarum* strains, namely LQC 2320 and LQC 2520, which exhibited proteolytic, lipolytic and antibacterial capacities, as well as three *Wickerhamomyces anomalus* strains, namely LQC 10343, LQC 10353 and LQC 10360, which exhibited both proteolytic and antimould potential, were reserved for further research, including applications in sourdough breadmaking. Kuppusamy et al. [3] evaluated the probiotic potential of *Lp. plantarum* RJ1 and *Pediococcus pentosaceus* S22, isolated from Hanwoo steer cattle fresh rumen fluid, through a series of phenotypic tests, as well as their performance during alfalfa and crimson clover silage fermentation. The results obtained were quite satisfactory, revealing the great potential of these strains.

Finally, the manufacturing of products exhibiting specific characteristics was reported by Popovic et al. [4] and Campaniello et al. [5]. In the first study, the functional potential of yogurt produced using the autochthonous strains *Streptococcus thermophilus* BGKMJ1–36 and *Lactobacillus bulgaricus* BGVLJ1–21, previously isolated from artisanally prepared sour milk and yogurt, respectively, was evaluated [4]. For this purpose, the technological and health-promoting capacity of the strains was assessed, along with their capacity to survive simulated gastrointestinal track conditions and adhere to Caco-2 cells. The results obtained were very promising, because after survival under simulated GIT conditions, the strains were able to adhere to the Caco-2 cells and upregulate genes related to autophagy and epithelial barrier defense. Campaniello et al. [5] evaluated the potential of the functional *Lp. plantarum* 178, previously isolated from pork meat, to produce Italian fermented sausages under industrial conditions. The strain effectively drove fermentation, providing the desired technological characteristics and at the same time inhibiting pathogenic and spoilage bacteria. In addition, the capacity of the strain to provide higher acidification scores at 25–30 °C than the two commercially available strains that were also assessed and the ability to perform acidification, even at 15 and 44 °C, makes this strain a very interesting alternative from a technological perspective.
